# Deep learning in intracranial EEG for seizure detection: advances, challenges, and clinical applications

**DOI:** 10.3389/fnins.2025.1677898

**Published:** 2025-10-30

**Authors:** Wasi Ur Rehman Qamar, Min-Ho Lee, Berdakh Abibullaev

**Affiliations:** ^1^Department of Robotics, Nazarbayev University, Astana, Kazakhstan; ^2^Department of Computer Science, Nazarbayev University, Astana, Kazakhstan

**Keywords:** clinical neurophysiology, high frequency oscillations (HFO), intracranial EEG (iEEG), epileptiform discharges, machine learning in healthcare, deep learning, neural signal analysis, signal processing

## Abstract

Deep learning has emerged as a transformative tool for the automated detection and classification of seizure events from intracranial EEG (iEEG) recordings. In this review, we synthesize recent advancements in deep learning techniques including convolutional neural networks (CNN), recurrent neural networks (RNN) with long short term memory (LSTM) units, and transformer based architectures that enable accurate localization of epileptogenic zones (EZ) in drug resistant epilepsy. These approaches effectively extract spatial and temporal features from raw iEEG signals to detect epileptiform discharges (ED) including seizures alongside other electro-physiological biomarkers such as high-frequency oscillations (HFO). Importantly, beyond relying solely on these traditional markers, several studies have indicated direct seizure detection by modeling ictal and preictal dynamics. Such methods capture alternative biomarkers including spectral changes, connectivity patterns, and complex temporal signatures that directly reflect seizure activity. Although deep learning models often achieve high accuracy, they continue to face several challenges due to data scarcity, heterogeneity in iEEG acquisition, inconsistent preprocessing protocols, and limited model interpretability. We also highlight emerging integrative strategies that combine multimodal neuroimaging data with deep learning analyses as well as neuromorphic computing techniques designed for real-time clinical application. Addressing these limitations has significant potential for surgical planning, reducing diagnostic subjectivity, and ultimately enhancing patient outcomes in epilepsy care.

## 1 Introduction

Epilepsy is a major neurological disorder affecting approximately 65 million people worldwide, imposing a substantial global health burden (Dash et al., 2022; Milligan, 2021). Characterized by recurrent, unprovoked seizures, this chronic condition disrupts daily functioning and adversely impacts cognitive performance, psychosocial integration, and overall quality of life. Although anti-seizure medications (ASM) serve as the basis of treatment, about one-third of patients show drug resistant epilepsy (DRE) experiencing persistent seizures despite appropriate pharmacological intervention (Worrell and Gotman, 2011; Enatsu and Mikuni, 2016). Such people face limited therapeutic alternatives which leads to rising morbidity and highlights the necessity for alternative techniques. In cases of drug resistant epilepsy, surgical intervention can be curative, although its effectiveness depends on accurately identifying and completely resecting the epileptogenic zone (EZ), defined as the smallest cortical region whose removal yields seizure freedom (Frauscher, 2020; Jobst et al., 2020). Achieving this precision means finding a balance between increasing seizure control and avoiding disturbance of healthy functionally necessary brain tissue.

Traditionally, EZ localization has depended predominantly on expert visual inspection of intracranial electroencephalography (iEEG) recordings (Flanary et al., 2022; Sperling, 1997). Although this procedure is an established clinical standard, it requires considerable effort and expertise due to the size and complexity of the data. Moreover, the process can be highly subjective. Multiple studies have identified significant inter-expert variability in labeling iEEG patterns potentially leading to inconsistent outcomes in surgical planning and patient care (Flanary et al., 2022; Weiss et al., 2019). Such variability highlights the need for computational tools particularly deep learning (DL) methods that can reduce subjectivity, enhance reproducibility, and improve clinical decision making.

While automated seizure detection excels at identifying ictal events in long-term iEEG recordings reducing review time for clinicians, its primary clinical value lies in supporting the localization of the epileptogenic zone (EZ) and seizure onset zone (SOZ). The primary goal of epilepsy surgery is to identify the smallest cortical area whose removal eliminates seizures, while balancing effectiveness with the preservation of critical tissue (Nissen et al., 2021). Deep learning addresses this by extracting spatiotemporal patterns that associate detected events with EZ boundaries. Nevertheless, detection alone does not determine surgical margins.

In response, researchers have increasingly developed automated and semi-automated approaches to facilitate EZ localization (Bernabei et al., 2023a; Ahmedt-Aristizabal et al., 2017). Advances in computational neuroscience have accelerated this shift, showing the potential of deep learning in processing complex iEEG signals. Specifically, deep learning enables automated feature extraction from raw input data, thus showing the complex spatiotemporal dynamics of neural activity (Mirchi et al., 2022). Transformer based architectures in particular have started to gain attention for seizure detection and iEEG classification (Potter et al., 2022; Wang et al., 2021; Zhang X. et al., 2024; Sun et al., 2022; Yang et al., 2024) extending the capabilities of more traditional CNN and RNN based methods (Antoniades et al., 2017, 2016; Liu et al., 2019; Zuo et al., 2019; Ren et al., 2021; Lai et al., 2019).

Among the most notable applications of deep learning in this domain is the automated detection and classification of seizure events. Such events are believed to represent key pathophysiological processes leading to irregular neural activity and the formation and spreading of epileptic seizures. Their correlation with the epileptogenic zone has caused an increased focus on deploying deep learning techniques not only for accurate seizure identification but also for differentiating pathological signals from normal background patterns (Lai et al., 2019; Liu et al., 2016). Seizure signals show significant heterogeneity causing substantial difficulties for conventional signal processing methods. Modern deep learning architectures including transformer based networks provide an effective solution to these challenges by directly modeling complex temporal dependencies (Wang et al., 2021; Potter et al., 2022; Zhang X. et al., 2024). Although traditional methods using Long Short Term Memory (LSTM) networks or CNN have exceeded 90% accuracy in identifying epileptiform activity (Medvedev et al., 2019), emerging alternatives ranging from neuromorphic systems to hyperdimensional computing are also showing potential for real-time, low-power detection, potentially suitable for implantable surgical devices (Schindler and Rahimi, 2021; Burrello et al., 2018, 2019). Deep Learning not only detects events but simulates epileptogenicity indices (EI) by scoring channel contributions to EZ propagation, guiding resection margins and predicting postoperative seizure reduction (Yamamoto et al., 2021; Zhang et al., 2021).

**Figure 1:**
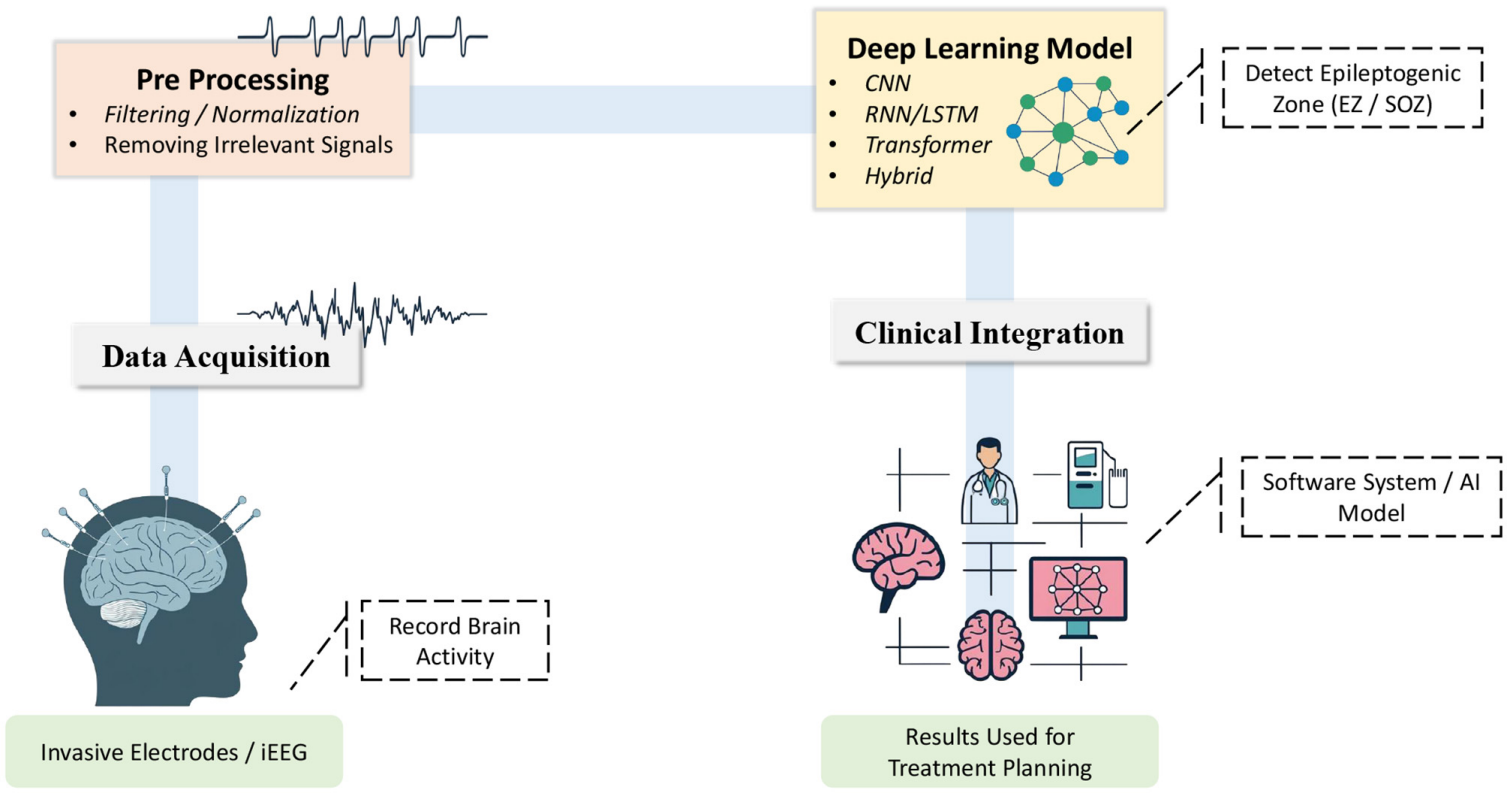
An overview of the workflow for detecting epileptogenic zones using deep learning. (1) **Data Acquisition:** Brain signals are recorded through electrodes. (2) **Preprocessing:** Noise is removed and signals are downsampled to retain clinically relevant information. (3) **Deep Learning Model:** A neural network architecture is applied to detect epileptogenic zones (EZ/SOZ). (4) **Clinical Integration:** The model results inform treatment planning enabling clinicians to utilize the identified zones to direct therapeutic actions.

Nevertheless, recent studies highlight the importance of standardizing automated seizure detection methods and performing strict clinical validation (Bernabei et al., 2023a; Abibullaev et al., 2010; Ahmedt-Aristizabal et al., 2017; Sindhu et al., 2020; Dimakopoulos et al., 2022). These initiatives highlight the challenges in selecting and validating appropriate algorithms for clinical use while also providing guidelines for safely integrating advanced computational methods into epilepsy surgery workflows. This review offers a structured examination of methodologies for seizure detection and classification including transformer based models, RNN, CNN, and emerging alternative architectures. We discuss the limitations of conventional iEEG analysis and automated approaches, propose potential solutions, and outline future research directions to strengthen clinical implementation and improve surgical planning outcomes. Figure 1 shows the whole workflow from iEEG acquisition to surgical decision making. The subsequent sections explore the significance of seizure detection (Section 3), review current deep learning strategies (Section 4), current challenges and ethical considerations (Section 5), and conclude by outlining prospective developments (Section 6).

## 2 Literature search methodology and strategy

A systematic literature review was undertaken to identify and analyze studies applying deep learning (DL) methods to seizure detection within the context of epilepsy surgery. The review specifically targeted peer-reviewed journal articles and conference proceedings that utlized intracranial EEG (iEEG) data to develop, evaluate, or clinically integrate DL based techniques. Comprehensive searches were conducted across multiple academic databases, including PubMed, IEEE Xplore, Scopus, and Web of Science using various keyword combinations coupled with Boolean operators focusing on literature published in the last five years. Principal search queries included:

- “Deep Learning” AND “Epilepsy Surgery”
- “Intracranial EEG (iEEG)” AND “Deep Learning”
- “Epileptiform” AND “Seizure” AND “iEEG” AND “Deep Learning”
- “High Frequency Oscillation (HFO)” AND “Epilepsy” AND “iEEG” AND “Deep Learning”

Additional targeted terms such as “CNN”, “RNN”, “LSTM”, “Transformer”, and “Neuromorphic Computing” were also used to refine the scope. Articles referenced within the bibliographies of selected studies were subsequently examined to ensure a comprehensive collection of relevant literature.

**Figure 2:**
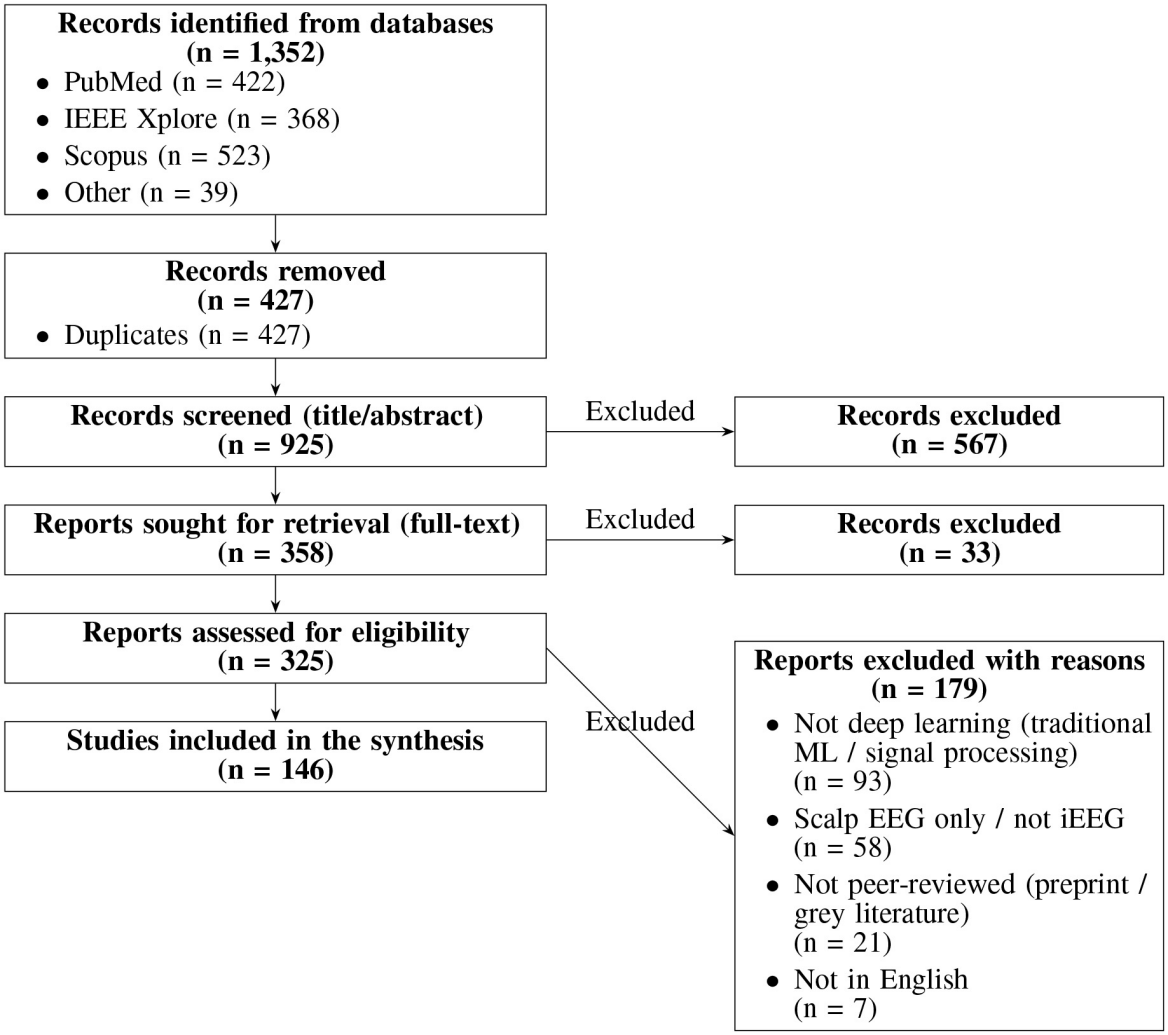
PRISMA flow diagram illustrating the study selection process for the systematic review.

The screening and selection process followed explicit inclusion and exclusion criteria (Figure 2) and involved multiple stages. Initially, titles and abstracts were inspected to exclude clearly unrelated works. Next, the full texts of potentially relevant papers were examined to confirm their alignment with the review objectives. Finally, only those that met all inclusion criteria were retained for detailed comparative analysis. From each selected study, key methodological and outcome details were extracted including the size and characteristics of the dataset, the type of deep learning architecture utilized (CNN, RNN/LSTM, Transformer based models), and performance metrics such as accuracy, sensitivity, specificity, or area under the curve (AUC). This structured approach enabled a thorough synthesis of recent advancements, ongoing methodological challenges, and future research prospects in deep learning based seizure analysis for epilepsy surgery.

## 3 Electrophysiological biomarkers in epilepsy

High frequency oscillations (HFO) which are considered a distinct type of epileptiform discharge (ED) along with other ED patterns are important electrophysiological biomarkers in current epilepsy research and clinical practice (Krikid et al., 2023; Smith et al., 2022).

HFO, typically defined as transient events in the 80–500 Hz range, are thought to result from synchronized neuronal firing within the epileptogenic zone (EZ) (Worrell and Gotman, 2011; Kobayashi et al., 2017). These oscillations can appear during interictal and ictal periods and are usually classified into ripples (80–250 Hz) and fast ripples (250–500 Hz) (Jacobs et al., 2012; Lee et al., 2019; Ye et al., 2023). More evidence suggests that HFO rich regions overlap significantly with the EZ making these oscillations valuable indicators for localizing seizure onset zones in surgical candidates (Zhang et al., 2021; Qing et al., 2024).

An ongoing challenge in applying HFO to clinical practice involves distinguishing pathological HFO (pHFO) from physiological HFO (pHO) (Cimbalnik et al., 2018). Both share similar frequency ranges although originate from different underlying processes (Weiss et al., 2019; Zhang et al., 2022). Table 1 summarizes the commonly used morphological (e.g., amplitude, duration) and spectral (e.g., power distribution, phase amplitude coupling) features utilized to differentiate pHFO from pHO (Weiss et al., 2019; Sindhu et al., 2024; Lai et al., 2020). This differentiation is essential for ensuring that surgical resections target only epileptogenic tissue thereby minimizing the risk of normal cognitive functions.

**Table 1:** Morphological and spectral distinctions between pathological and physiological HFO.

**Features**	**Pathological HFO (pHFO)**	**Physiological HFO (pHFO)**	**References**
**Qualitative Characteristics**			
Frequency range	Fast ripples: 250–500 Hz	Ripples: 80–250 Hz	Jacobs et al., 2012; Lee et al., 2019
Temporal association	High temporal coupling with epileptiform spikes	Little or no spike association	Charupanit et al., 2020
Amplitude	High amplitude and strong contrast to background	Lower amplitude relative to background	Qing et al., 2024
Spectral characteristics	Broader spectral profile with complex phase amplitude coupling	Narrower spectral content with typical PAC patterns	Lai et al., 2020
Localization	Predominantly localized to the epileptogenic zone (EZ)	Distributed across normal functional regions	Zhang et al., 2021; Qing et al., 2024
**Quantitative Measures [values from Matsumoto et al. (** **2013** **)]**			
Spectral amplitude (z-score)	9.07 ± 3.49	4.10 ± 0.65	
Mean frequency (Hz)	188.4 ± 104.7	264.2 ± 162.1	
Duration (ms)	23.1 ± 19.5	12.1 ± 8.3	

**Figure 3:**
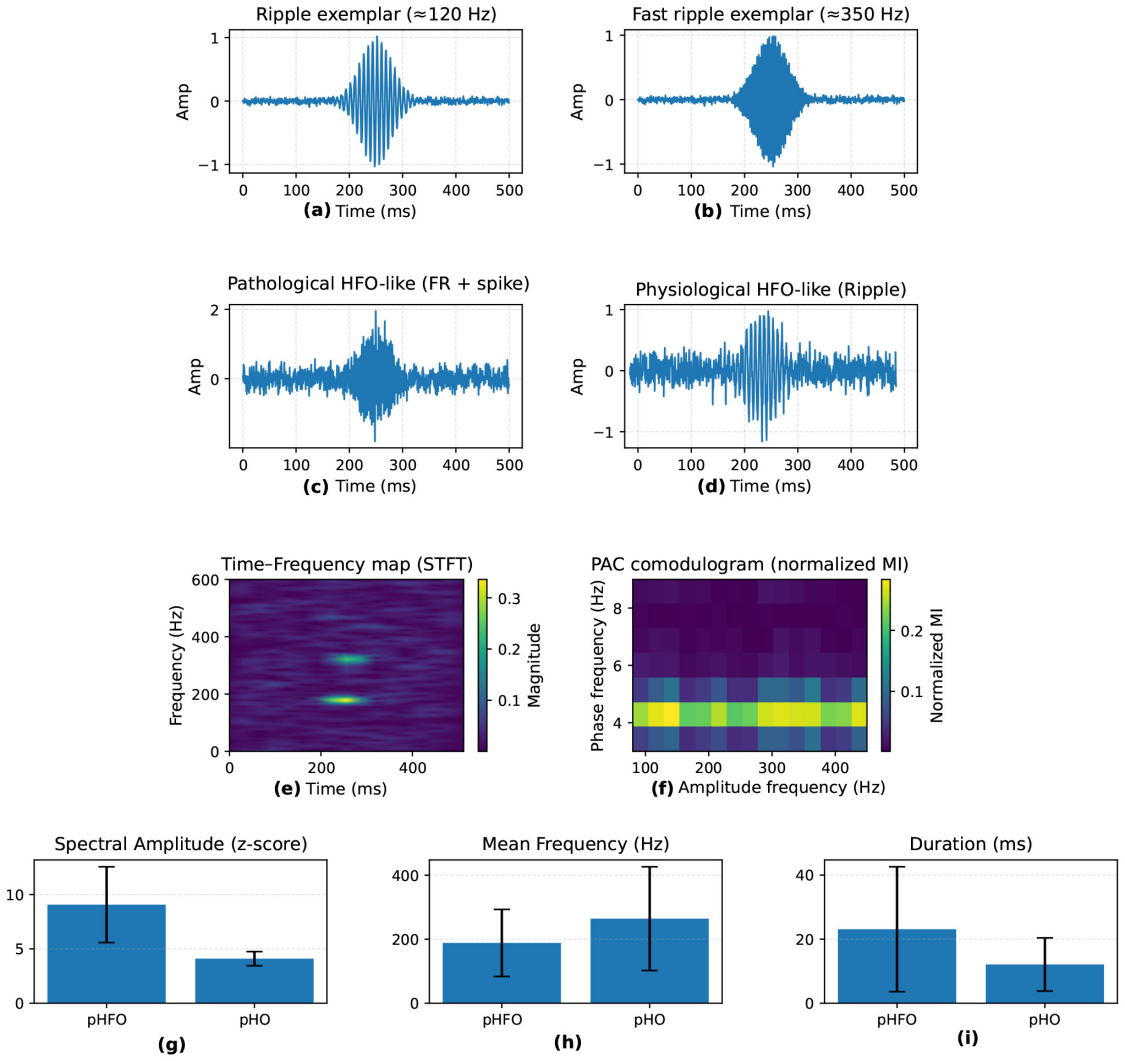
Visual summary of HFO in epilepsy. **(a, b)** Time-domain examples of ripples (80–250 Hz) and fast ripples (250–500 Hz). **(c, d)** Pathological HFO (pHFO) show spike association and higher amplitude, while physiological HFO (pHO) appear in normal networks. **(e)** Time-frequency map with transient HFO bursts; **(f)** phase-amplitude coupling (PAC). **(g–i)** Quantitative feature comparisons (spectral amplitude, mean frequency, duration).

To complement the quantitative summary in Table 1 and Figure 3 provides a visual overview of how high-frequency oscillations (HFO) are characterized in epilepsy research. It highlights typical ripple and fast ripple waveforms, contrasts pathological and physiological HFO morphologies, and shows their expression in time–frequency space and through phase—amplitude coupling (PAC). The lower panels summarize key quantitative metrics reported in the literature, including spectral amplitude, mean frequency, and event duration, which help distinguish pathological from physiological activity.

The EZ includes the seizure onset zone (SOZ; early ictal involvement), regions prominent in high-frequency oscillations (HFO; indicative of hypersynchronous firing), and irritative zones (interictal spike generators that may extend beyond the SOZ) (Cai et al., 2021). Deep learning identifies epileptogenic zones (EZ) by integrating them through multi-task models: for instance, convolutional neural networks (CNN) extract spatial high-frequency oscillation (HFO) patterns, while transformers assess the temporal propagation of the seizure onset zone (SOZ) in relation to irritative activity, producing channel-specific scores (e.g., through attention maps) that identify resectable tissue. This reduces qualitative variability in visual inspection; however, validation against postoperative outcomes is important for clinical confidence (Broti et al., 2025).

Epileptiform discharges (ED) including spikes, sharp waves, and spike-and-wave complexes are likewise fundamental to understanding epilepsy pathophysiology and guiding treatment strategies (Zhao et al., 2018; Jaoude et al., 2019; Kural et al., 2020). ED generally present as temporary high amplitude waveforms (approximately 20–70 ms in duration) sometimes followed by slow-wave components (Falach et al., 2024). These events are examined in terms of morphological, temporal, and spectral properties to determine their clinical significance. Table 2 outlines qualitative and quantitative measures that assist in differentiating pathological ED from benign variants (Zhao et al., 2018; Yamamoto et al., 2021). Although manual iEEG analysis remains a clinical benchmark, emerging automated tools utilizing deep learning show potential in reducing manual work and lead to more consistent evaluations (Kolodziej et al., 2023; Zhao et al., 2022b).

**Table 2:** Morphological and quantitative distinctions between pathological and benign epileptiform discharges.

**Features**	**Pathological ED**	**Benign variants**	**References**
**Qualitative Characteristics**			
Waveform morphology	Sharp high amplitude spikes or sharp waves with a steep onset and a pronounced after slow component	Less defined lower amplitude transients that often lack a distinct after slow	Tjepkema-Cloostermans et al., 2024; Janmohamed et al., 2022
Temporal association	Often occur in clusters or display periodicity aligning with epileptogenic activity	Typically isolated events without consistent clustering	Tong et al., 2024; Tveit et al., 2023
Amplitude contrast	Significant difference toward the background (peak amplitudes well above baseline)	Lower amplitude relative to the background within normal variation	Tjepkema-Cloostermans et al., 2024; Tong et al., 2024
Localization	Predominantly focal (mapping to the epileptogenic zone) or generalized in specific syndromes	More diffusely distributed with no clear focal origin	Janmohamed et al., 2022
After-slow component	Clear after-slow deflection following the spike indicating post-ictal suppression	Typically absent or minimal with a smoother return to baseline	Janmohamed et al., 2022
**Quantitative Measures**			
Spike duration (ms)	Spikes: 20–70, Sharp Waves: 70–200	Generally shorter or more variable (often less distinct < 50)	Da Silva Lourenço et al., 2021; Tong et al., 2024
ED rate	Often increased (commonly 2–4 discharges per minute or higher)	Rare or sporadic in normal recordings (typically < 0.5/min)	Tong et al., 2024; Tveit et al., 2023

Overall, reliable detection and differentiation of HFO and ED are essential for refining epilepsy diagnostics and surgical interventions. Advances in computational neuroscience and large scale data analytics continue to drive improvements in the accuracy, consistency, and clinical relevance of these electrophysiological biomarkers.

## 4 Deep learning for detection of HFO, epileptiform discharge, and seizure dynamics

Traditional machine learning and signal processing techniques have significantly advanced automated seizure detection, particularly in identifying high-frequency oscillations (HFO) and epileptiform discharges (ED) (Abibullaev et al., 2009; Weiss et al., 2019; Sharifshazileh et al., 2021; Burelo et al., 2022; Burrello et al., 2019; Schindler and Rahimi, 2021; Krikid et al., 2021; Lachner-Piza et al., 2019; Guo et al., 2021; Sciaraffa et al., 2020; Sindhu et al., 2020; Dimakopoulos et al., 2022; Wong et al., 2020; Donos et al., 2020; Sindhu et al., 2024; Li et al., 2021b). However, these approaches face persistent challenges. They typically depend on manual feature extraction and extensive parameter tuning which limit robustness and generalizability across heterogeneous iEEG datasets. In addition, balancing sensitivity and specificity often requires complex post-processing or careful recalibration for different seizure biomarkers.

Recent advances in deep learning (DL) represent a paradigm shift by enabling end-to-end feature learning directly from raw iEEG signals (Hoogteijling and Zijlmans, 2021). Unlike traditional methods that rely on manual features, DL architectures learn rich, nonlinear representations that can capture complex seizure related patterns including HFO, ED, and subtler biomarkers (Wang et al., 2021). Various architectures have been explored, including convolutional neural networks (CNN), recurrent neural networks (RNN), and more recently transformer based models which can effectively model the spatio-temporal dynamics of epileptic events (Geng et al., 2021; Singh and Malhotra, 2022; Wang et al., 2024b; Ma et al., 2018; Zuo et al., 2019; Ma et al., 2019; Sadek et al., 2023; Liu et al., 2019; Zhao B. et al., 2020; Ren et al., 2021; Zanghieri et al., 2021; Wang et al., 2020; Abderrahim et al., 2023). Transformers are particularly promising due to their self-attention mechanisms which model long-range dependencies and contextual relationships while supporting parallel processing (Guo et al., 2022; Vaswani et al., 2017). To improve clinical applicability, several studies have proposed lightweight CNN and transformer networks optimized for patient-independent real-time seizure detection (Si et al., 2023; Yang et al., 2024; Sun et al., 2022; Potter et al., 2022). Hybrid frameworks that combine CNN with RNN or transformers aim to utilize each model strengths, CNN for spatial representation and RNN or transformers for temporal dependencies resulting in more robust and adaptable detection systems (Ouichka et al., 2022; Sun L. et al., 2024).

Recent DL approaches have also focused on localizing the epileptogenic zone (EZ) through probabilistic mapping. Hybrid CNN-LSTM models, for instance, assign EZ likelihood scores to individual channels by linking HFO/ED density with seizure onset zone (SOZ) propagation, often analyzed via Granger causality (Wang et al., 2024b). Attention mechanisms further refine these models by reducing the influence of non-propagating spikes, generating saliency heatmaps to guide surgical inspection (Gunnarsdottir et al., 2022; Nejedly et al., 2025). Transformer based models extend the classic epileptogenicity index (EI) by learning dynamic, channel-wise margins: multi-head attention layers capture HFO-SOZ correlations, while adaptive thresholds minimize irritative zone spillover (Yamamoto et al., 2021). Such techniques enable predictive modeling of surgical outcomes in drug-resistant epilepsy by identifying interictal epileptogenic networks, guiding resections to maximize overlap with these networks and reduce distance from resected tissue. Reported performance reaches an AUC of 0.85-0.86 for predicting ≥50% seizure reduction and Engel Class I/II outcomes (Partamian et al., 2025). Key challenges remain in validating these models for multifocal epilepsy where resection margins are inherently ambiguous.

Beyond HFO and ED, DL has expanded to capture ictal dynamics directly, offering insight into seizure onset and progression critical for surgical planning (Ma et al., 2018; Singh and Malhotra, 2022; Dwaraka et al., 2021; Sun Y. et al., 2024; Zhao X. et al., 2020; Zhang and Zhou, 2023). For example, time-aware RNN effectively model the temporal evolution of ictal states (Palanichamy and Ahamed, 2022) while autoencoder based frameworks have distinguished focal from non-focal epilepsy biomarkers (Jangde et al., 2023). Advanced time-frequency methods such as spectral envelope analysis further enrich representations by characterizing power changes during seizures (Zhang and Zhou, 2023). Ensemble models that integrate CNN, SVM, and LSTM outputs have achieved superior accuracy by combining complementary strengths (Usman et al., 2021). Other hybrid designs for example, reconstruction independent component analysis (R-ICA) combined with LSTM networks reduce computational complexity while maintaining performance (Dwaraka et al., 2021).

**Figure 4:**
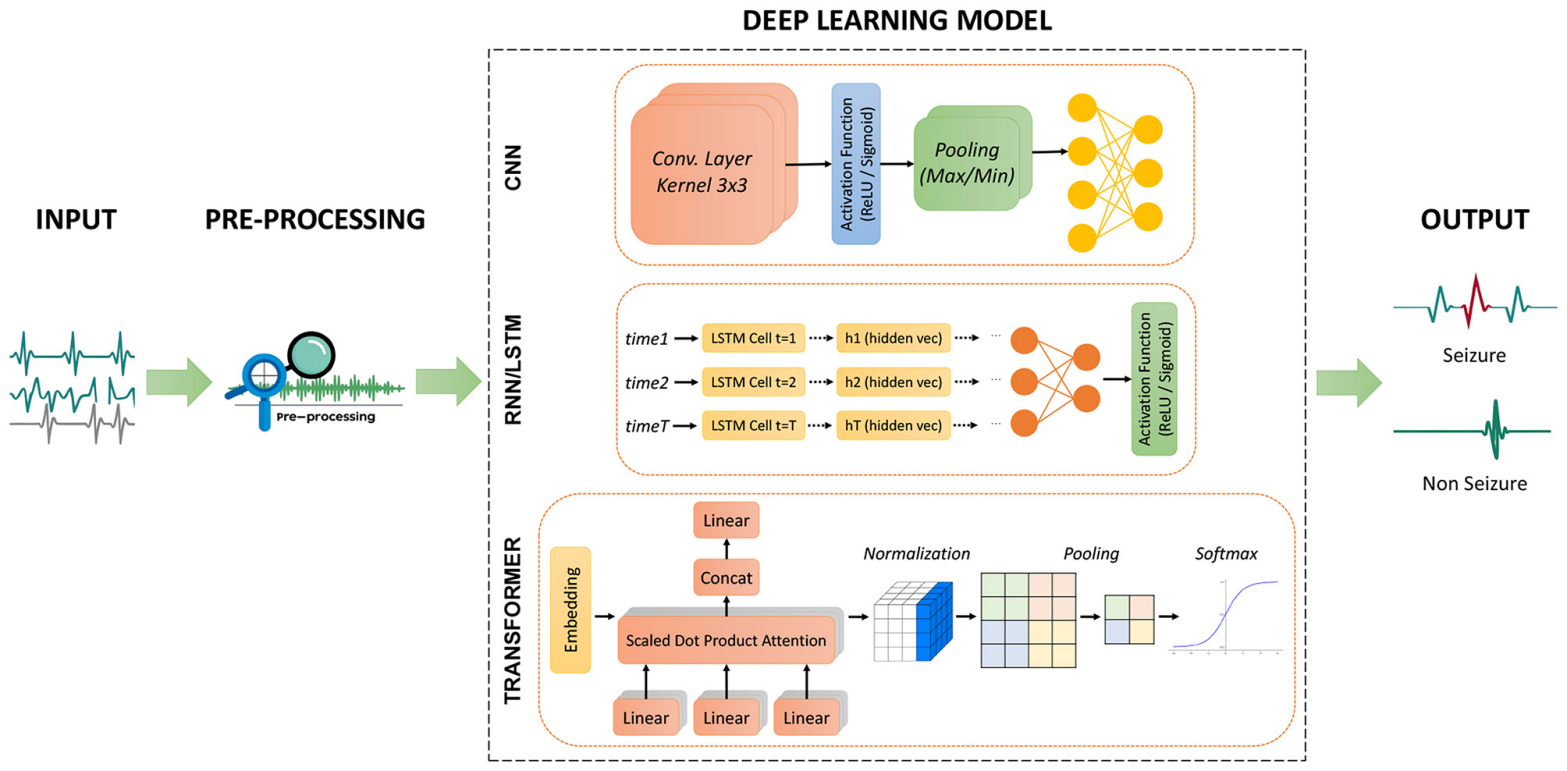
Overview of three deep learning architectures for seizure detection and classification. **CNN:** Convolutional layers extract features from raw signals, followed by activation, pooling, and fully connected layers for classification. **RNN/LSTM:** Sequential modeling of preprocessed iEEG captures temporal dependencies before final classification. **Transformer:** Self-attention layers model long-range dependencies followed by normalization and softmax classification.

Finally, DL based architectures contribute to EZ/SOZ localization by clustering high-density biomarkers (e.g., HFO during seizures) to specific channels, thereby supporting surgical decision-making (see Section 5). Figure 4 summarizes common deep learning paradigms for seizure detection and classification, illustrating how CNN, RNN/LSTM, and transformers are utilized to model the complex spatio-temporal structure of iEEG.

### 4.1 CNN: advanced architectures for spatiotemporal feature extraction

CNN have gained significant attention for their efficacy in analyzing iEEG data due to their capacity for extracting both spatial and temporal features (Lai et al., 2019; Ren et al., 2021; Zuo et al., 2019). By stacking convolutional and pooling layers, CNN can learn complex representations directly from raw signals. These representations are important for detecting HFO, ED, and related biomarkers essential to localizing the epileptogenic zone (EZ). Additionally, the high sensitivity and specificity that CNN based models achieved make them suitable for real-time clinical applications. Many recent studies have refined CNN based algorithms for seizure detection and classification as summarized in Tables 3, 4.

**Table 3:** Overview and performance comparison of CNN based deep learning architectures for seizure detection and classification.

**References**	**Model architecture**	**Performance metrics**	**Other metrics**	**Study highlights**
**High frequency oscillations**				
(Lai et al. 2019)	STE-based detection; deep 2D CNN on CWT time-freq. maps	**Sensitivity** Ripples 88.16%, Fast Ripples 93.37% **Specificity** 91.52%	Prec 88.67%; Rec 91.27%; F1 89.95%; FDR: Ripples 12.6%, FR 8.1%	Improves HFO detection with high sensitivity and low false discovery
(Sadek et al. 2023)	Pretrained ResNet-18 features; dimensionality reduction + k-means clustering	–	–	Unsupervised HFO clustering to aid seizure onset localization
Zhao B. et al. (2020)	RMS threshold + Morlet scalograms; pretrained ResNet101 (transfer learning)	**Accuracy** Artifacts ~99%, Spikes ~98%, Ripples ~96%, FR ~97%	Sen 94.4% (10 dB) / 97.2% (15 dB); Prec 97.1/97.2%; F1 94.4/97.2%; AUC up to 0.988	Integrated detection, classification, and mapping of HFO for presurgical evaluation
(Zuo et al. 2019)	1D → 2D grayscale; CNN blocks with dropout + FC layers	Ripples: HFO 93.1% / non 88.1%; FR: HFO 88.4% / non 93.4%; Sen: Ripples 77.0% / FR 83.2%; Spec: Ripples 72.3% / FR 79.4%	Cohen κ 0.54/0.78; Spearman 0.86/0.94 (p < 0.01); Proc: 20s / 5 min signal	Enhanced HFO detection with better sensitivity and specificity
(Ma et al. 2019)	CNN on 714 × 714 CWT scalograms; two conv + pooling + FC layers	Rec 89.37%; Prec 94.19%; F1 91.71%	Detection 1-3s / event; Training ~3 min	Efficient HFO detection with high precision and recall
(Ren et al. 2021)	CNN on four 2D image types (orig., filtered, wavelet, ASPWVD); conv, pool, FC	Acc: Ripples 80.9 ± 1.4%, FR 77.9 ± 1.6%; Sen: 80.1 ± 2.0%, 77.4 ± 2.5%; Spec: 82.5 ± 2.3%, 78.8 ± 2.3%	–	Differentiates epileptogenic vs non-epileptogenic HFO
(Zhao et al. 2022b)	Three models: SVM; FCNN (2 × 32); CNN (dense 128); Adam optimizer	Acc: STFT/CNN 88.1; Entropy/FCNN 80.1; Entropy/FCNN/PU 76.9	–	Localizes SOZ from 20 s iEEG segments; PU learning reduces labeling effort
Zhang Y. et al. (2024)	Two CNN: artifact rejection + spkHFO detection; uses 128 × 128 time-freq./amp. maps	STE: Art Acc 98.6 ± 0.2%, spkHFO 89.2 ± 0.8%; MNI: Art 98.7 ± 0.6%, spkHFO 89.8 ± 3.1%; Sens: STE Art 99.2 ± 0.3, spkHFO 90.8 ± 1.9; MNI Art 94.8 ± 2.7, spkHFO 90.9 ± 4.2	STE: F1 99.1 ± 0.1, Prec 99.0 ± 0.3; spkHFO F1 91.4 ± 0.6, Prec 92.1 ± 2.1; MNI: F1 90.2 ± 4.9, Prec 100; spkHFO F1 94.1 ± 1.9, Prec 97.8 ± 0.7	Automated HFO detection/classification using STE & MNI with CNN

**Table 4:** Overview and perfromance comparison of CNN based deep learning architectures for seizure detection and classification.

**References**	**Model architecture**	**Performance metrics**	**Other metrics**	**Study highlights**
**Epileptiform Discharges**				
(Antoniades et al. 2016)	- CNN1 (Shallow : One convolutional layer and FC sigmoidal layer) - CNN2 (Deeper : Two convolutional layers and FC layer)	**Accuracy** Temporal : 77.20% TF : 86.67% CNN1 : 79.48% CNN2 : 87.51%	**Filter Correlations** First layer : 42% Second layer : 52% McNemar test : p < 0.01	Automatically extracts features from the time domain for ED detection
(Liu et al. 2019)	- Stockwell transform to generate time frequency representations - Deep 15 layer CNN with dropout and batch normalization	**Sensitivity** Segment Based : 97.01% Event Based : 95.45% **Specificity** Segment based : 98.12%	**False Discovery Rate** 0.36 per hour	Achieves high sensitivity and specificity in automatic seizure detection
(Yamamoto et al. 2021)	- Processes raw iEEG signals via temporal convolutional layers with ReLU - Final sigmoid output layer	**AUC** 0.944 ± 0.067 (using EpiNet) Sensitivity : 0.878 ± 0.143 Specificity : 0.933 ± 0.072	**SVM AUC** 0.808 ± 0.253 **Preictal vs Early Seizure AUC** Epi-Net : 0.910 ± 0.107 SVM : 0.804 ± 0.194 **Epileptogenicity Index AUC** 0.822 ± 0.135	Detects the early phase of epileptic seizures from iEEG signals
(Zhao et al. 2022a)	- Eight layer 1D CNN - Three convolutional layers - Kernel size 1 × 10 - Filters : 32, 64, 32 - Max pooling (1 × 5, stride 4) - Fully connected layer	**EEGAug (Patient 1)** Accuracy : 97.55% Recall : 51.89% Specificity : 99.95% **CB Focal Loss (Patient 1)** Accuracy : 97.32% Recall : 51.00% Specificity : 99.75%	**Patient 1 Precision** EEGAug : 98.42% CB Focal Loss : 92.23% **F1 Score** EEGAug : 67.38% CB Focal Loss : 65.30% **Matthews Correlation Coefficient** EEGAug : 70.22% CB Focal Loss : 67.27% **Cohen Kappa** EEGAug : 66.26% CB Focal Loss : 64.03%	Classifies invasive iEEG signals to localize the epileptic focus by bypassing manual feature extraction and using a novel DCT based data augmentation method
(Antoniades et al. 2017)	- Four 1D convolutional layers - Flattening, fully connected layer - Logistic regression with ordered score labels	**Accuracy Multiclass CNN** ~3% improvement w.r.t binary AUC : 0.900	40 - 50 × higher computational cost but 14% improvement in accuracy over alternatives	Detects interictal epileptiform discharges in iEEG with improved sensitivity and balanced false positives
(Jaoude et al. 2019)	- Three convolutional layers - Kernel sizes : 32, 16, 8 samples - Max pooling, dropout - Batch normalization - FC layer with sigmoid output	AUC : 0.996 ± 0.002 **Sensitivity** *False Positive Rate (< 6/min)* 97.0 ± 1.0% *False Positive Rate (< 1/min)* 84.0 ± 4.0% at < 1 *Positive Predictive Value (0.80)* 67.7 ± 11.0%	**Precision Recall Curve** AUCPR : 0.807 ± 0.066 Partial AUC (Specificity>0.9) 0.981 ± 0.006	Detects mesial temporal lobe epileptiform discharges in iEEG from foramen ovale electrodes
(Li et al. 2019)	−21 layer 1d CNN - Kernel size 3 - Max pooling, dropout - Batch normalization - Softmax output	Accuracy : 85.14% True Positive Rate : 88.76% True Negative Rate : 81.68%	False Positive Rate : 18.32% False Negative Rate : 11.24% Precision: 82.26% F1 Score: 85.39%	Automates epileptic focus localization using iEEG recordings without preprocessing or manual feature extraction.

#### 4.1.1 HFO detection via CNN

In a study, Ma et al. (2019) proposed a CNN based framework for HFO detection in iEEG by converting 80 - 500 Hz signals into time-frequency scalograms. Trained on 18,000 labeled samples, their five-layer CNN achieved 94.19% precision. Sadek et al. (2023) proposed an unsupervised clustering framework using a temporal basis set, dimensionality reduction, and a deep cluster module to separate HFO from artifacts and ripple peaks, successfully identifying the seizure onset zone (SOZ) for potential surgical guidance. Zhao B. et al. (2020) developed a model combining bandpass filtering, amplitude thresholding, and time-frequency scalograms, classified by four ResNet101 CNN into artifacts, spikes, ripples, and fast ripples. Similarly, Zuo et al. (2019) transformed 1D iEEG signals into 2D grayscale images and used a pyramidal CNN to classify ripples and fast ripples from non-HFO.

Furthermore, Zhao et al. (2022b) extended CNN for SOZ localization by segmenting iEEG into 20s intervals, extracting spectral and entropy features, and comparing classifiers (CNN, SVM, FCNN). Using positive-unlabeled learning to limit labeling needs, PU learning achieved 76.91% with only 15.87% labeled data. More recently, Ren et al. (2021) proposed a multi-stream CNN that processes raw and filtered waveforms, wavelet spectrograms, and smoothed pseudo Wigner-Ville distributions to distinguish EZ from non-EZ HFO. Lai et al. (2019) similarly combined short time energy (STE) estimation with a CNN architecture using filters of various sizes. Expanding on these advances, Zhang Y. et al. (2024) introduced PyHFO, an open-source platform with CNN based classifiers and fast detection algorithms to distinguish artifacts from true HFO and separate spike-associated from non-spike HFO. Tested on iEEG datasets, PyHFO achieved up to 50x faster performance than MATLAB tools like RIPPLELAB.

#### 4.1.2 Epileptiform discharge analysis via CNN

Antoniades et al. (2017) introduced a CNN based approach for detecting ED. By relying on continuous score labels rather than binary classifications, they improved both sensitivity and the ability to capture salient ED morphologies thereby enhancing clinical interpretability. In an earlier study, Antoniades et al. (2016) showed that CNN could effectively learn features for subject independent ED classification training on data from 25 patients without manual feature engineering. Similarly, Jaoude et al. (2019) achieved area under the curve (AUC = 0.996) in detecting mesial temporal lobe ED using a CNN trained on large augmented datasets from 46 patients.

In related work, Li et al. (2019) presented a one dimensional CNN that classifies focal versus non-focal signals directly from raw iEEG recordings achieving 85.14% accuracy. Meanwhile, Liu et al. (2019) combined Stockwell transform (S-transform) with a 15-layer CNN achieved high sensitivity (97.01%) and specificity (98.12%) across a public database of 21 patients. Yamamoto et al. (2021) developed EpiNet, a CNN architecture that outperformed an SVM baseline with 0.944 AUC, and introduced a data driven epileptogenicity index (d-EI) correlating with surgical outcomes. Further simplifying the approach, Zhao et al. (2022a) proposed a one dimensional CNN that operates directly on raw iEEG data combining a novel discrete cosine transform (DCT) based data augmentation method to address limited labeled data achieving high classification performance on the Juntendo iEEG dataset.

### 4.2 RNN and LSTM: modeling temporal dependencies

RNN have an important role for modeling the complex temporal dependencies of iEEG signals with LSTM architectures being particularly effective in addressing seizure detection challenges (Medvedev et al., 2019). By maintaining internal states and combining of memory cells through feedback loops, RNN offer a robust mechanism to capture transient neural events including HFO and ED (Rezk et al., 2020). LSTM networks, a specialized variant of RNN, minimize the problem of vanishing gradients through gating mechanisms (input, forget, and output gates) thus preserving both short and long term temporal dependencies (Lindemann et al., 2021). Table 5 present an overview of RNN/LSTM based architectures for seizure detection across different iEEG biomarkers.

**Table 5:** Overview and performance comparison of RNN/LSTM based deep learning architectures for seizure detection and classification.

**References**	**Model architecture**	**Performance metrics**	**Other metrics**	**Study highlights**
**High frequency oscillations**				
(Liu et al. 2021)	Bi-branch network: – Branch1: 1D ResNet + LSTM on 80-500 Hz signals – Branch2: 2D ResNet + CBAM on time-freq images Fused outputs → MLP	Acc: Intra 93.62%, Cross 89.76% Sen: Intra 94.62%, Cross 92.00% Spec: Intra 92.70%, Cross 88.26%	**Intra:** Prec 92.1%, F1 93.3%, FDR 7.9%, SEN_SPE 93.6% **Cross:** Prec 86.9%, F1 89.1%, FDR 13.1%, SEN_SPE 89.9%	Automated HFO detection aiding epileptogenic zone localization
**Epileptiform Discharges**				
(Medvedev et al. 2019)	Bidirectional LSTM (200 units) on spectral power from 8 bands over 3 time bins	Acc >90% in all cases Sen < 86% in only 2 cases Spec >90% overall	–	Detects epileptiform spikes/HFO with high within- and between-subject accuracy
(Geng et al. 2021)	GRU classifier (IEDnet) + AC-GAN for synthetic augmentation	**DS1:** Acc 93.4%, Sen 93.2%, Spec 93.5% **DS2:** Acc 93.1 ± 3.2%, Sen 89.2 ± 9.9%, Spec 95.3 ± 2.3%	**DS1:** F1 90.4%, FDR 12.1%, FPR 6.5%, AUROC 93.4% **DS2:** F1 89.7 ± 5.1%, FDR 9.0 ± 3.6%, FPR 4.7 ± 2.3%, AUROC 92.3 ± 4.5%	Detects epileptiform discharges in iEEG with data augmentation
**Other Epileptic Seizure Dynamics**				
(Wang et al. 2024b)	GRU network estimating effective connectivity via Granger causality + graph analysis	Acc: Group O Max 6/7 (85.7), Some 5/7 (71.4); Group P Max 4/5 (80), Some 3/5 (60)	–	Localizes epileptogenic zones using directional iEEG interactions
(Kolodziej et al. 2023)	1D iEEG → LSTM(20) → two FC → Softmax	Acc 98%; Sen 100%; Spec 99%	Prec 96%	Captures temporal dynamics in iEEG with high detection accuracy
(Wang et al. 2021)	Dual branch: – Classical feats (time, freq., nonlinear) → Bi-LSTM + attn – Raw iEEG → 1D CNN; fused → MLP	Acc: Bern 97.6, Bonn 92.1, SEEG Intra 92.5, Cross 90.3 Sen: Bern 97.8, Bonn 91.1, Intra 93.2, Cross 89.1 Spec: Bern 97.4, Bonn 93.0, Intra 91.8, Cross 91.6	Test time 40 ms/signal	Combines classic signal processing and DL for SEEG
(Palanichamy and Ahamed 2022)	Time-aware CNN for spatio-temporal features + LSTM	Acc 88.6%; Rec 90.9%	Prec 87.7%; F1 89.2%	Automated seizure detection with denoising, z-score norm, TA-CNN + LSTM
(Dwaraka et al. 2021)	Two-stage: preprocess/segment → RICA features → LSTM(100/125/100)	Acc 98.9%; Sen 99.0%; Spec 98.7%	BalAcc 99.2%; F1 98.3%	RICA + LSTM for effective temporal learning in iEEG
(Wang et al. 2024a)	Dual CNN-LSTM (two 1D conv streams, BN, MP, LSTM); concat + GAP + FC	*Event:* Sen – Freiburg 100, SWEC 100, AES 90.5 *Segment:* Acc – Freiburg 93.5, SWEC 99.6, AES 78.2; Sen – 88.1/99.1/69.2; Spec – 94.0/99.7/79.9	*Event FPR (h^−1^):* Freib 0.10, SWEC 0, AES 0.47 *Segment AUC:* Freib 0.910, SWEC 0.994, AES 0.737	Channel ranking by per-ch F1; incremental subset selection for prediction
(Singh and Malhotra 2022)	FFT-based spectral feats → CNN → LSTM	Acc 94.7%; Sen 95.8%; Spec 94.5%	AUC 95.1%; F1 94.8%; Test 50 ms	Real-time preictal seizure prediction

#### 4.2.1 HFO and ED analysis via RNN/LSTM

In a study, Liu et al. (2021) proposed a hybrid bi-branch neural network for HFO detection, combining a 1D ResNet-LSTM on bandpass-filtered signals with a 2D ResNet-CBAM on wavelet transforms. A multilayer perceptron integrated these features, achieving high sensitivity, specificity, and accuracy in both intra and cross subject tests. In another study, Geng et al. (2021) presented IEDNet which integrates LSTM, GRU, and an auxiliary classifier GAN to detect interictal epileptiform discharges (ED). Synthetic data augmentation further boosts detection robustness. Medvedev et al. (2019) similarly demonstrated the efficacy of bi-directional LSTM networks in capturing multiple forms of epileptiform activity including spikes and ripples achieving overall accuracies exceeding 90%.

#### 4.2.2 Seizure dynamics modeling via RNN/LSTM

Wang et al. (2021) proposed a multi-branch fusion network with a Bi-LSTM and attention mechanism, utilizing bidirectional temporal context and feature weighting to outperform single direction LSTM in distinguishing epileptogenic from non-epileptogenic signals. Kolodziej et al. (2023) combined wavelet based higher order statistics and chaos theory measures with an LSTM classifier achieving seizure detection accuracies exceeding 98%. Singh and Malhotra (2022) proposed a frequency band decomposition method that feeds iEEG signals into both CNN and LSTM architectures for seizure prediction. Wang et al. (2024b) utilized Gated Recurrent Units (GRUs) within a Granger causality framework to localize ictal onsets and analyze network connectivity patterns in iEEG data.

Similarly, Wang et al. (2024a) introduced a dual CNN-LSTM model that utilizes a channel reordering approach achieving 100% event based sensitivity on two standard iEEG datasets. Other hybrid systems include TA-CNN-RNN (Palanichamy and Ahamed, 2022) where a time-aware CNN integrates spatial and temporal feature extraction before passing outputs to an LSTM based classifier achieving accuracies of 88.6% on the Bonn dataset. Dwaraka et al. (2021) combined Reconstruction Independent Component Analysis (RICA) with LSTM layers (RICA-LSTM) attaining an accuracy of 98.92%. These results collectively highlights the capacity of LSTM based models to precisely capture short and long-term dependencies in iEEG data thereby facilitating more reliable detection of diverse seizure biomarkers. RNN and LSTM have shown their effectiveness in identifying temporal patterns in iEEG recordings enhancing accuracy in detecting HFO, ED, and other seizure related events. Although modern LSTM implementations are sufficiently optimized for potential real-time applications, further work remains necessary to optimize deployment in resource constrained clinical environments particularly where on-device processing or ultra-low-latency inference is critical.

### 4.3 Transformer models: capturing long-range temporal context

Transformer based architectures originally developed for natural language processing (Vaswani et al., 2017; Kumar and Khan, 2023; Anwar et al., 2024) are increasingly being adopted in biomedical signal analysis for their capability to model extensive temporal dependencies in parallel. Unlike RNN which process data sequentially, transformers utilize a multi head self-attention mechanism to capture complex relationships across entire sequences supported by positional encodings that preserve temporal order (Lin et al., 2022). These properties are particularly advantageous for seizure detection where ictal events and interictal patterns may span variable durations and occur at multiple time scales.

The success of transformer models for seizure analysis comes from key architectural advantages. First, multi-head attention enables the model to attend to different time-scale dependencies concurrently (Li et al., 2021a). Second, positional encodings ensure that sequence order is effectively maintained during parallel processing (Li et al., 2023). Third, feed-forward layers within transformer blocks facilitate complex, nonlinear transformations of attention weighted representations (Gillioz et al., 2020). Table 6 summarizes recent developments in transformer based frameworks for iEEG analysis.

**Table 6:** Overview and performance comparison of transformer based deep learning architectures for seizure detection and classification.

**References**	**Architectures**	**Performance**	**Other metrics**	**Study highlights**
(Potter et al. 2022)	Autoencoder with input projection, learnable pos. encoding, multihead self-attn, geometric masking	Acc: MIT 0.87 ± 0.006, UPenn 0.68 ± 0.014, TUH 0.61 ± 0.009 Rec: MIT 0.90 ± 0.006, UPenn 0.76 ± 0.013, TUH 0.57 ± 0.009	MIT: Prec 0.98 ± 0.003, AUC 0.94 ± 0.023 UPenn: Prec 0.88 ± 0.01, AUC 0.73 ± 0.027 TUH: Prec 0.92 ± 0.005, AUC 0.57 ± 0.013	Anomaly detection: train on non-seizure data; high reconstruction error flags seizures
(Sun et al. 2022)	Shared CNN for feature extraction + transformer encoders; channel-wise mixup for variable iEEG channels	Event-based Sen: SWEC-ETHZ 97.5%, TJU-HH 98.1%	FDR (false alarms/hr): SWEC 0.06, TJU 0.22 Latency: SWEC 13.7 s, TJU 9.9 s Inference: 30 min/128ch < 1 s	Subject-independent seizure detection using CNN temporal feats + transformer spatio-temporal patterns
(Yang et al. 2024)	CNN branch + conv embedding + alternating transformer blocks (context & spatial)	Cross-subj AUROC: FNUSA 0.97, Mayo 0.93 Cross-inst AUROC: 0.89, 0.89	Cross-subj AUPRC: FNUSA 0.76, Mayo 0.87 Cross-inst AUPRC: 0.81, 0.79	Classifies iEEG into artifact/pathologic/physiologic; supports EZ localization in drug-resistant epilepsy
(Yu et al. 2023)	1D CNN for local feature extraction + transformer encoder w/ causal conv multihead attn	–	Improved AUROC & AUPRC	iEEG classification for epilepsy diagnosis; outperforms prior models
Sun Y. et al. (2024)	Hybrid CNN-transformer: channel embedding, multihead self-attn, MLP classifier	Acc: SWEC 91.15%, HUP 88.84% Sen: SWEC 89.48%, HUP 87.41% Spec: SWEC 92.82%, HUP 90.27%	SWEC: AUC 0.9700, Prec 93.0%, F1 90.8% HUP: AUC 0.9463, Prec 91.1%, F1 86.9%	Continuous seizure detection on long-term iEEG with high sens. & low latency
(Jiang et al. 2024)	Wavelet packet decomposition → CNN embedding → transformer (freq. weighting) → HMM for seizure phase	Acc 92.75%; Rec 92.06%; Spec 94.59%	Prec 96.31%	Detects & phases temporal lobe epilepsy via signal processing + attention + temporal state modeling

In a study, Potter et al. (2022) developed an unsupervised anomaly detection approach using transformers, training an encoder on non-seizure data and identifying seizures through enhanced reconstruction errors. Sun et al. (2022) introduced a combined Transformer-CNN method for raw multichannel iEEG data. Similarly, Yang et al. (2024) utilized a hierarchical structure which combines local features (extracted by CNN) with global representations (extracted by transformer layers) enhancing classification performance. Yu et al. (2023) presented IEEG-CT which integrates a 1D CNN and a transformer encoder with convolutional multi-head attention outperforming prior models.

Further advancements include Sun Y. et al. (2024) proposed a subject-independent seizure detection model combining channel-wise mixup with multi-task transformer learning, achieving high AUC and improving generalization across patients and electrode setups. Jiang et al. (2024) extended this concept by merging wavelet packet decomposition with a convolution based embedding module and transformer blocks in a hybrid HMM-Wavformer design achieving 92.75% seizure detection accuracy while identifying crucial frequency bands.

Transformer based methods showed an exceptional ability to capture extensive contextual information in iEEG recordings while preserving critical temporal patterns (Wang et al., 2021). Despite these advancements, other deep learning architectures such as CNN and LSTM remain competitive particularly when combined with advanced signal representations. Ongoing research thus focuses on hybrid architectures, optimizing real-time performance and enhancing clinical interpretability. Comparisons indicate that transformer based models frequently outperform conventional CNN and RNN on long-sequence tasks (Potter et al., 2022; Sun et al., 2022; Yang et al., 2024; Yu et al., 2023) further emphasizing their potential for real-time seizure detection where parallel processing and precise segmentation of critical neurological events is important.

### 4.4 Alternative deep learning architectures

Although CNN, RNN, and transformer based methods dominate the area of deep learning for seizure detection, several alternative architectures have also gained attention by providing unique benefits and methodological innovations (Wu et al., 2021; Li et al., 2022; Jangde et al., 2023). These architectures utilize autoencoders, variational approaches, or graph based neural networks to capture complex iEEG patterns beyond the capabilities of more conventional techniques. By focusing on unsupervised or semi-supervised learning structures, they often reduce reliance on large labeled datasets while maintaining robust performance.

Li et al. (2022) introduced a convolutional variational autoencoder (CVAE) based approach for unsupervised HFO detection. In their method, time-frequency maps of candidate HFO (ripples and fast ripples) were derived from continuous wavelet transforms with only the red channel retained to highlight dominant frequency components. Similarly, Wu et al. (2021) proposed a similarly multi-stage strategy. First, they identified events of interest using a Hilbert envelope based threshold, converting these segments into time-frequency representations via an optimized wavelet transform (SE-CMWT). They then utilized a stacked denoising autoencoder (SDAE) to extract robust features before classifying events with an AdaBoost based SVM ensemble enhanced by sample weight adjusting factors (SWAF-ABSVM). Gharebaghi and Sardouie (2024) also applied advanced signal modeling to HFO detection by converting raw iEEG segments into graph structures. Each time sample was treated as a node, embedding energy-based features (RMS, short-time energy) within a learnable adjacency matrix. A Deep Graph CNN then classified HFO events, reporting a sensitivity of 90.7%. Table 7 summarizes these diverse architectures and their respective outcomes in seizure detection tasks.

**Table 7:** Overview and performance comparison of alternate deep learning architectures for seizure detection and classification.

**Reference**	**Architectures**	**Performance**	**Other metrics**	**Study highlights**
(Li et al. 2022)	Convolutional variational autoencoder: conv encoder/decoder; latent space clustered via k-means	Acc 92.85%; Sen 93.91%; Spec 92.14%	Latent dim opt: 30 dims → overall 92.96% 40 dims → Spec 97.36%	Unsupervised HFO detector bypassing manual feature design
(Wu et al. 2021)	Time-freq maps (Shannon entropy + Morlet) → AdaBoost SVM ensemble	Sen: Ripples 92.4%, FR 90.3%	FDR: Ripples 9.2%, FR 10.7%	Automated HFO detection from iEEG to aid EZ localization
(Gharebaghi and Sardouie 2024)	iEEG segments → graph (time samples = nodes) → Deep Graph CNN + 1D CNN + dense classifier	Sen 90.7 ± 1.26%; Spec 93.3 ± 1.36%	AUC 0.96	Graph-based temporal representation with DGCNN for automated HFO detection
(Li et al. 2022)	Conv. variational autoencoder with encoder/decoder; latent space clustered via k-means	Acc 92.85%; Sen 93.91%; Spec 92.14%	Latent dim opt: 30 dims → overall 92.96% 40 dims → Spec 97.36%	Unsupervised HFO detector avoiding manual feature design
(Wu et al. 2021)	Time-freq maps (Shannon entropy + Morlet) → AdaBoost SVM ensemble	Sen: Ripples 92.4%, FR 90.3%	FDR: Ripples 9.2%, FR 10.7%	Automated HFO detection from iEEG aiding epileptogenic zone localization
(Gharebaghi and Sardouie 2024)	iEEG segments → graph (time samples as nodes) → Deep Graph CNN + 1D CNN + dense layers	Sen 90.7 ± 1.26%; Spec 93.3 ± 1.36%	AUC 0.96	Graph-based temporal representation with DGCNN for automated HFO detection

While many deep learning studies focus on seizure detection, it is important to distinguish this from the clinically critical task of EZ/SOZ localization. Seizure detection primarily assists in efficiently screening long-term iEEG recordings, whereas localization links pathological patterns such as HFO and ED to their spatial and temporal distributions, which directly informs surgical planning. Thus, CNN, RNN/LSTM, and Transformer based approaches should not only be viewed as tools for automated detection but also as additional techniques that help in identifying epileptogenic regions for resection.

**Figure 5:**
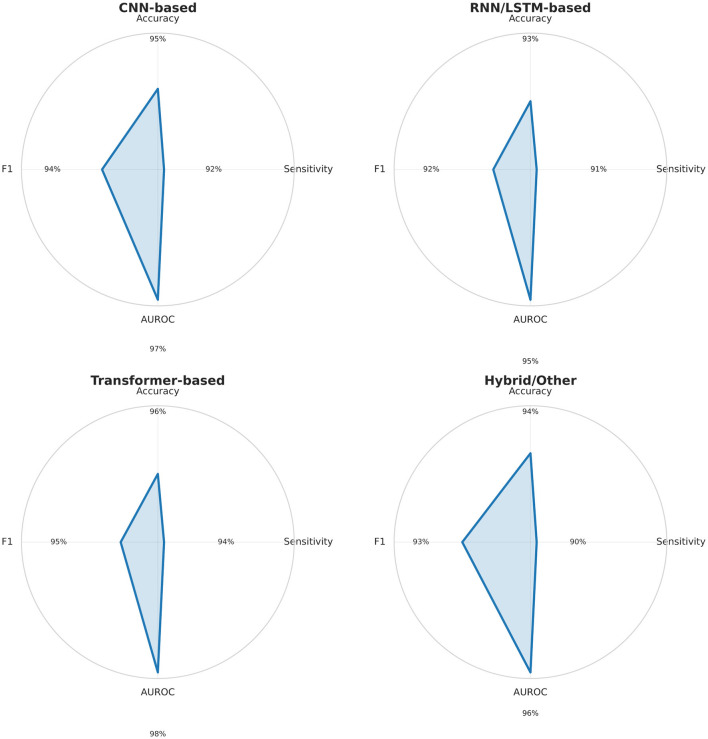
Radar charts summarizing Accuracy, Sensitivity, AUROC, and F1-score (0–100%) for major deep learning model families (CNN, RNN/LSTM, Transformer, Hybrid) based on Tables 3–7. CNN and Transformer models show balanced, high performance; RNN/LSTM models excel in sensitivity but vary in AUROC; hybrid methods remain heterogeneous.

To complement the detailed tables, Figure 5 presents radar charts summarizing the performance profiles of the main deep learning model families (CNN, RNN/LSTM, Transformers, and hybrid architectures). This visualization enables a quick comparison of accuracy, sensitivity, AUROC, and F1-score across methods. CNN show strong and well-balanced performance; RNN/LSTM achieve high sensitivity but struggle with cross-patient generalization; Transformers combine high accuracy with strong AUROC, indicating better scalability; hybrid models remain promising but inconsistent. These patterns provide a clear, visual guide for selecting architectures and designing future seizure detection systems.

## 5 Methodological challenges and limitations

Despite the potential of deep learning in automating seizure detection, the field currently faces several methodological challenges including data availability, preprocessing protocols, and technical constraints. Such challenges affect model robustness, generalizability, and clinical adoption. Key among these issues are limitations in dataset size and representativeness, lack of standardized preprocessing pipelines, data scarcity and heterogeneity, and the complexity of deep learning algorithms which complicates model interpretability. Table 8 summarizes these fundamental barriers.

**Table 8:** Methodological challenges in deep learning based seizure detection and classification methods.

**Challenge**	**Description**
**Non-invasive localization (Jeong et al.**, **2022****; Makaram et al.**, **2023****)**	Localizing the epileptogenic zone solely via non-invasive modalities remains challenging. Advanced deep learning methods for multimodal integration are emerging to improve localization accuracy.
**Manual detection of HFO (Sharifshazileh et al.**, **2021****; Dimakopoulos et al.**, **2024****; Burelo et al.**, **2022****; Zhang et al.**, **2021****)**	Manual HFO detection is time-consuming and prone to inter-rater variability. Distinguishing pathological HFO from physiological ones remains a critical challenge for reliable clinical adoption.
**Clinical and data heterogeneity (Bernabei et al.**, **2023a****)**	Variability in patient characteristics, including epilepsy subtypes, electrode implantation methods, and treatment approaches, introduces substantial heterogeneity. Underlying pathologies further complicate the generalizability of deep learning models.
**Robust iEEG-based detection (Zhang X. et al.**, **2024****)**	Noise artifacts, non-stationary signals, and data imbalance reduce detection reliability. Reproducibility concerns and limited cross-patient generalizability persist, limiting widespread clinical translation.

### 5.1 Preprocessing and feature extraction

Studies in seizure detection frequently utilizes various signal preprocessing and feature extraction methodologies which can lead to inconsistent or non-replicable findings (Bernabei et al., 2023a; Zhang X. et al., 2024). These inconsistencies become visible in diverse artifact removal practices, variance in filtering parameters, and signal normalization schemes each of which can significantly affect model performance (Bernabei et al., 2023a; Zhang X. et al., 2024). Consequently, comparing results across different research groups or replicating methodologies becomes challenging as different methods obscure whether performance gains arise from genuine algorithmic improvements or insignificant differences in data handling.

The issue extends beyond basic signal conditioning to include feature selection. Different studies may choose distinct sets of signal characteristics (e.g., temporal, spectral, or higher order measures) thereby affecting detection rates and challenging cross-validation efforts (Bernabei et al., 2023a; Zhang X. et al., 2024). As such, establishing standardized protocols for preprocessing and feature extraction remains a high priority. Initiatives that develop and provide comprehensive recommendations similar to standardized frameworks in other areas of biomedical signal processing are essential for sustaining meaningful comparisons, ensuring reproducibility, and ultimately advancing the reliability of deep learning based seizure detection. Table 9 describes best practices for data harmonization and standardized preprocessing pipelines to enhance reproducibility in iEEG based deep learning studies.

**Table 9:** Recommended best practices for reproducible iEEG preprocessing in deep learning studies.

**Step**	**Best practice and rationale**
Data harmonization	Adopt the **BIDS-iEEG** standard for metadata (e.g., electrode labels, patient demographics) to ensure interoperability and minimize dataset-specific biases in multi-center studies.
Artifact removal	Use automated techniques such as **ICA** or **RPCA** to remove non-neural artifacts (e.g., muscle activity, eye blinks).
Filtering	Apply a **Standard bandpass filter** (0.5–500 Hz) with **Notch filtering** (50/60 Hz); use ripple-specific filtering (80–250 Hz) for HFO related tasks.
Normalization and segmentation	Perform **per-channel z-score normalization** and use fixed-length windowing (e.g., 1–5 s epochs) for model input consistency.
Validation and transparency	Report all preprocessing parameters in supplementary materials and use **open-source tools** (e.g., MNE-Python) to share code and pipelines for reproducibility.

### 5.2 Data scarcity and heterogeneity

Data scarcity and heterogeneity present significant challenges to developing robust, generalizable deep learning models (Bernabei et al., 2023a; Zhang X. et al., 2024; Keutayeva and Abibullaev, 2024). Despite significant progress having been made in releasing more comprehensive iEEG datasets, publicly available resources still tend to be small and lack uniform annotation and quality control protocols. The problem is made worse by the high variability in recording conditions, patient demographics, and seizure types.

In clinical practice, epilepsy takes multiple forms and requires different electrode placements and recording methodologies. As a result, data from different sources show significant heterogeneity, complicating cross-institutional model evaluations (Bernabei et al., 2023a). Moreover, limited data often lead to overfitting wherein models perform better during training but fail to generalize effectively across broader patient data. Tables 10, 11 detail the inconsistencies in sample size, annotation quality, and public accessibility among the commonly utilized iEEG datasets. Addressing these limitations will require continuous community wide collaboration, including the development of larger, more diverse datasets and the adoption of shared annotation protocols.

**Table 10:** Comparison of datasets used in deep learning based seizure detection and classification methods.

**References**	**Dataset**	**Pts**	**Type**	**Rate (Hz)**	**Annotation Protocol**	**Public**
(Burrello et al. 2019)	SWEC-ETHZ (Long Term)	18	iEEG	1,024	Visual review by epileptologist for seizure on/offset; artifact channels removed	Yes
(Burrello et al. 2018)	SWEC-ETHZ (Short Term)	13	iEEG	512	Visual review; artifacts removed; 3 min preictal/ictal/postictal included	Yes
(Karoly et al. 2018)	Melbourne NeuroVista	12	iEEG (ECoG)	400	Onset at 1 min; offset 10 s before recording end	Yes
(Fedele et al. 2017)	iEEG-1 (CRCNS)	20	iEEG	4,000	Automated HFO (ripples, fast ripples) detection using validated algorithm	Yes
(Andrzejak et al. 2012)	Bern-Barcelona	5	iEEG	512	Two electroencephalographers labeled “focal” vs “nonfocal” channels by visual review	Yes
Bbrinkm et al. (2014)	UPenn & Mayo Seizure Challenge (Canine)	5	iEEG	400	1 s clips labeled “Ictal”; interictal ≥1 h from seizure; durations matched to seizure length	Yes
(Singh and Malhotra 2022)	UPenn & Mayo Seizure Challenge (Human)	2	iEEG	3,000	1 s clips labeled “Ictal” (full seizure + latency); interictal ≥1 h from any seizure	Yes
(Bernabei et al. 2023b)	HUP (Hosp. Univ. Pennsylvania)	57	ECoG / SEEG	512–1,024	Clinical seizure onset channels + resected/ablated zones documented	Yes
(Andrzejak et al. 2001)	Bonn Univ.	5	iEEG	173.61	23.6 s segments after artifact review; screened for weak stationarity	Yes
(Frauscher et al. 2018)	Montreal Neurological Inst.	106	iEEG (SEEG)	200–2,000	Non-epileptogenic cortex, awake (eyes closed); 60 s artifact-free segments selected	Yes
(Janca et al. 2014)	Spike Detector (CVUT)	30	iEEG	200–1,000	Spikes manually marked by 3 neurophysiologists; kept if ≥2 agreed	Yes
(Maiwald et al. 2004)	Univ. Freiburg	21	iEEG	256–512	Seizure onsets marked by epileptologists; in-focus/out-of-focus electrodes chosen	Yes

**Table 11:** Comparison of datasets used in deep learning based seizure detection and classification methods.

**References**	**Dataset**	**Pts**	**Type**	**Rate (Hz)**	**Annotation Protocol**	**Public**
(Jaoude et al. 2019)	Massachusetts Gen. Hosp./Harvard Med.	46	iEEG (FO)	1,024	Epileptologist labeled “definite/indeterminate” ED via GUI; ≥250 ED/patient; spikes in 250 ms windows	No
(Yamamoto et al. 2021)	Osaka Univ. Hosp.	21	iEEG	10,000	Onset/interictal visually marked by 2 epileptologists; cross-checked with video, self-reports, records	No
(Li et al. 2021c)	Rensselaer Polytech. Inst. (RPI)	19	iEEG	512	Onset/offset labeled by epileptologist and independently verified	No
(Nejedly et al. 2018)	FNUSA (St. Anne's Univ. Hosp.)	11	iEEG	25,000	Manual SignalPlant labels; 3 s epochs classed phys./pathol./50-60Hz/noise/artifacts (power/raw review)	No
(Nejedly et al. 2018)	Mayo Clinic	25	iEEG	32,000	SignalPlant labels; 3 s epochs incl. 60Hz noise; expert envelope/raw scoring	No
(Liu et al. 2021)	Xuanwu Hosp., Capital Med. Univ.	5	iEEG (SEEG)	2,048	Manual visual marking by two clinical experts	No
(Cook et al. 2013)	Private	15	iEEG	400	NeuroVista staff + investigators; seizures by diaries, audio, auto detection	No
(Wang et al. 2024b)	Private	1	iEEG	256	Preictal/ictal/postictal segmentation; ictal further split (1–3) by expert/time criteria	No
(Geng et al. 2021)	Univ. of Miami	5	iEEG	256–1,024	Reviewed by 2 EEG experts; most active channel per patient marked by neurologist	No
(Medvedev et al. 2019)	AUH (Augusta Univ. Health)	5	iEEG	500	Auto threshold detection (8 bands) + expert verify; ground truth: 1,000 events/class (spikes/ripples etc.)	No
(Zhao et al. 2022a)	Epilepsy Ctr., Juntendo Univ.	6	iEEG	2,000	Expert annotation; sleep, motion-free ≥1h from seizures; SOZ/non-SOZ via visual + clinical review	No
(Ren et al. 2021)	Beijing Haidian Hosp.	19	iEEG	2,000	HFO auto-detect (5-min interictal); EZ confirmed via surgery + MRI/CT + Engel I (≥2y)	No
(Constantino et al. 2021)	NeuroPace	22	iEEG (RNS)	250	Ictal patterns by neurophysiologist; onset confirmed by epileptologist (cursor mark)	No
(Si et al. 2023)	TJU-HH (Tianjin Univ. Huanhu Hosp.)	12	iEEG (SEEG)	2,048	Full visual review by board-certified epileptologist; on/offset marked; artifacts removed	No
(Li et al. 2022)	West China Hosp.	5	iEEG	2,560	4042 time-freq maps manually labeled by 2 neuroelectrophysiologists via MATLAB GUI	No

### 5.3 Interpretability and explainability

A critical concern for the clinical adoption of deep learning based seizure detection systems is their black-box nature (Zhang et al., 2021). While such models can achieve high accuracy, the complex decision making processes limit clinical trust and acceptance. This challenge has started discussions about model agnostic versus architecture specific interpretability strategies (Stiglic et al., 2020). Model agnostic methods such as local surrogate models or feature importance scores offer broad applicability but may lack the granularity of architecture specific approaches which provide deeper insights by utilizing essential network components (e.g., attention weights, filter visualizations).

To address these limitations, several explainable AI (XAI) techniques have been systematically applied to deep learning models in iEEG analysis to enhance the interpretability of seizure detection and epileptogenic zone (EZ) localization (Patil and Patil, 2023). Gradient-weighted Class Activation Mapping (Grad-CAM) generates class-specific heatmaps by weighting convolutional layer activations with gradients from the target class, highlighting critical regions in input signals such as high-amplitude spikes in iEEG recordings associated with epileptic seizures (Kolodziej et al., 2023).

However, a central interpretability challenge lies in how models integrate multiple electrophysiological biomarkers into a unified EZ representation. While CNN can capture spatial HFO patterns and transformers highlight temporal propagation within the SOZ, the implicit weighting of SOZ, HFO, and irritative activity often remains opaque (Sun Y. et al., 2024; Yi et al., 2025). Attention maps and probabilistic EZ heatmaps partially address this issue but without explicit fusion strategies, clinicians may struggle to reconcile model outputs with established EZ definitions. Validation against postoperative outcomes therefore becomes essential to ensure clinical trust in such designations.

Recent findings highlight that combining multiple biomarkers such as pathological patterns alongside baseline activity can create more accurate insights into epileptogenic regions (Ye et al., 2023). This highlights the need for interpretable systems capable of contextualizing detection decisions based on these diverse signal elements. Although reverse engineering methods, including visualization and saliency mapping represent initial steps (Zhang et al., 2021), more advanced approaches that can explain how deep networks synthesize multi-modal iEEG information remain an active area of research. Achieving clinically relevant interpretability not only involves computational innovation but also collaborative input from clinicians, ensuring that model explanations align with established medical knowledge and practical decision making processes.

### 5.4 Technical details and hardware constraints

Implementing deep learning for seizure detection in real-world clinical environments imposes strict requirements on both data acquisition and computational infrastructure. First, sampling rates must be sufficiently high to capture the rapid transient events often characterizing seizure onset or HFO (Lee et al., 2019). While moderate sampling frequencies (e.g., 500 Hz to 1 kHz) may be sufficient for slower seizure activities, detecting higher frequency phenomena (e.g., fast ripples at 250–500 Hz) typically requires sampling rates exceeding 2 kHz, and very high frequency oscillations (VHFO) require sampling above 5 kHz (Lee et al., 2019). Lower sampling rates increase the possibility of noise and the potential loss of crucial temporal details thereby reducing the model accuracy and reliability.

Second, deploying deep learning models in real-time clinical settings is challenged by limited hardware resources and the energy constraints related to neuromorphic and embedded platforms (Xu et al., 2025; Schuman et al., 2022; Van Albada et al., 2018). These systems often have restricted memory footprints and computational throughput, necessitating careful optimization of network architectures (Li and Meng, 2023). Techniques such as model quantization and custom architecture design can reduce computational cost and memory usage while maintaining robust detection performance (Liang et al., 2021). Consequently, the choice of hardware platform combined with algorithmic efficiency remains critical for achieving real-time seizure detection and classification in clinical practice.

## 6 Future directions

Ongoing advances in deep learning for iEEG analysis have greatly expanded the range of computational tools available for epilepsy diagnosis and surgical planning. Addressing persistent methodological challenges such as robust algorithmic performance, model interpretability, and standardization of evaluation remains a priority. Table 12 outlines the open questions and future research directions in the field of deep learning based seizure detection.

**Table 12:** Future research directions and open questions in deep learning based seizure detection and classification methods.

**Research area**	**Open questions and future directions**
Data collection	How can multicenter collaborations produce larger, standardized datasets, including diverse epilepsy subtypes and recording paradigms?
Preprocessing	Which standardized filtering, artifact removal, and normalization protocols ensure reproducibility across institutions and hardware setups?
Model interpretability	Which novel visualization or explainable AI (XAI) techniques e.g., EI-like scores for resection margin guidance and postoperative outcome prediction (seizure freedom via survival models) can clarify deep learning decisions and improve clinician trust in automated seizure detection and EZ localization?
Ethical and equitable deployment	How can we address data privacy, bias, and cost barriers to ensure wide adoption, especially in low-resource settings?

One promising research focus lies in predictive modeling and outcome predictions. Although current deep learning systems can reliably detect and classify seizures, they increasingly aim to predict them and guide surgical interventions. By examining diverse iEEG biomarkers including HFO, ED, and connectivity metrics models can identify diagnostic signals associated with either favorable or unfavorable surgical outcomes (Zhang et al., 2021; Qing et al., 2024; Dimakopoulos et al., 2024). This capacity to predict postsurgical outcomes is especially valuable for refining patient selection and customizing resection strategies as shown by Dimakopoulos et al. (2024) who reported significant gains in seizure freedom rates when the epileptogenic zone was fully removed.

Multimodal data integration represents an additional approach for improving the sensitivity and specificity of automated seizure detection and localization. Incorporating MRI, fMRI, and MEG data provides a more deeper insight on the cortical and subcortical networks that cause seizure generation (Jeong et al., 2022; Makaram et al., 2023). However, the integration of multimodal data and neuromorphic computing introduces several practical limitations that must be addressed for widespread clinical adoption. High-resolution neuroimaging modalities such as fMRI and MEG are resource intensive with significant costs associated with acquisition equipment and specialized personnel, limiting accessibility in low-resource or rural settings (Ooi et al., 2025). Moreover, the computational demands of fusing heterogeneous data streams iEEG with volumetric MRI or dynamic fMRI require substantial processing power, potentially necessitating cloud based infrastructure that raises data privacy concerns and latency issues in real-time applications. Neuromorphic hardware while promising for low-power edge computing in implantable devices remains in early stages with limited scalability, high development costs, and compatibility challenges with existing clinical workflows (Sun et al., 2025). Future efforts should prioritize cost-effective hybrid solutions such as federated learning for distributed multimodal training to overcome these challenges and enhance equitable deployment. As these innovations move closer to clinical implementation, ethical, practical, and equity concerns demand careful attention. Ensuring patient privacy in the era of cloud based analytics is essential while issues such as algorithmic bias and uneven data representation can adversely impact diagnostic outcomes for specific demographic groups (Singh et al., 2023; Fong et al., 2024). Efforts to expand data diversity and establish transparent, fair model training protocols must align with ongoing initiatives to standardize clinical validation. Moreover, cost-effectiveness analyses, regulatory pathways, and user acceptance among clinicians and patients are all crucial elements in determining whether new AI driven solutions will be widely adopted.

Table 13 summarizes several ethical and clinical considerations important for effective integration of deep learning in epilepsy care. Ultimately, the way forward necessitates establishing closer relationships between engineers, clinicians, and other stakeholders. By coordinating strict technical development with robust patient centered evaluation, the next generation of deep learning based seizure detection and localization tools can be helpful in enhancing results for individuals with drug resistant epilepsy.

**Table 13:** Clinical implications and ethical considerations in deep learning based seizure detection and classification methods.

**Aspect**	**Key points and considerations**
Clinical efficacy and outcome prediction (Burelo et al., 2022; Dimakopoulos et al., 2024; Zhang et al., 2021; Qing et al., 2024)	Deep learning-based seizure analysis shows high accuracy in localizing epileptogenic zones and predicting outcomes. Complete resection of seizure-generating regions correlates with improved seizure freedom.
Regional access and infrastructure barriers (Fong et al., 2024)	Severe regional disparities in access to iEEG remain. Non-institution-centric training, better resource allocation, and cost-effective solutions are needed.
Ethical, transparency, and safety considerations (Singh et al., 2023; Zhang et al., 2021)	Deep learning models must be validated, explainable, and fair. Data privacy is critical. Clinicians require clear, interpretable predictions, minimal black-box behavior, and robust consent protocols.
Clinical validation (Lee et al., 2021; Kerr et al., 2024; FDA, 2021)	Prospective multi-center trials and FDA approvals are vital to validate deep learning efficacy, addressing patient variability and electrode coverage limitations for clinical integration.
Enhanced localization through multimodal integration (Jeong et al., 2022; Makaram et al., 2023)	Combining iEEG with multimodal MRI or fMRI can improve epileptogenic zone localization and reduce surgical uncertainties.

## 7 Discussion and conclusion

In this study, we reviewed recent advances in deep learning for the analysis of intracranial EEG (iEEG) data, focusing on seizure detection and epileptogenic zone (EZ) localization. We studied many different types of deep learning architectures including convolutional neural networks (CNN), recurrent neural networks (RNN), and transformer based models highlighting their ability to automate feature extraction and improve classification accuracy. Although deep learning architectures are proficient in seizure detection and offer temporal anchors for iEEG analysis, their clinical efficacy depends on the incorporation of localization metrics (such as HFO and SOZ mapping) to inform surgical decisions, as inaccurate EZ delineation may lead to incomplete resection or functional impairments. Additionally, we discussed the integration of hybrid models that combine CNN for spatial feature extraction with LSTM for temporal modeling enhancing the performance of seizure detection systems.

Despite these advancements, several challenges remain for the clinical adoption of deep learning based seizure detection systems. One of the primary limitations is the heterogeneity of the data as iEEG signals vary significantly between patients due to differences in electrode placement, acquisition protocols, and epilepsy pathology. The performance of deep learning models often declines when applied to unseen datasets, emphasizing the need for cross-institutional validation and improved generalization techniques.

Another critical challenge is the interpretability of deep learning models in clinical decision making. Unlike traditional machine learning approaches that rely on explicitly defined features, deep networks function as black-box models, making it difficult for clinicians to trust their predictions. Techniques such as Grad-CAM and saliency mapping have been explored to improve interpretability but further work is needed to ensure that these methods align with clinical expectations.

Realistically, deep learning can guide resectable areas by generating epileptogenicity index (EI) heatmaps that delineate SOZ and HFO rich regions from margin buffers, addressing localization ambiguity in epilepsy cases. By utilizing federated learning across multi-center datasets, these models predict postoperative seizure freedom aligning with the primary iEEG objective of optimizing surgical resection while minimizing functional deficits.

Additionally, real-time application limitations present an additional significant challenge. Many state-of-the-art deep learning models require substantial computational resources, making them impractical for real-time seizure monitoring in implantable or portable devices. Exploring lightweight architectures, neuromorphic computing strategies, and hardware optimization techniques could bridge this gap and facilitate real-time clinical deployment.

Future research should focus on improving the diversity and standardization of the data sets. The development of large-scale publicly available datasets that combine multiple recording environments, seizure types, and demographic variations is essential to improve model robustness. Transfer learning and domain adaptation techniques may also help reduce inter-subject variability and facilitate generalization across different datasets.

Another promising direction involves multi-modal data integration, combining iEEG with additional neuroimaging modalities such as functional MRI (fMRI), diffusion tensor imaging (DTI), and magnetoencephalography (MEG). These approaches have demonstrated potential in refining EZ localization by providing additional spatial and functional context to iEEG derived features. Furthermore, ethical and regulatory considerations surrounding deep learning in epilepsy care must be addressed. Ensuring data privacy, developing explainable AI frameworks, and establishing standardized clinical validation protocols are necessary steps for regulatory approval and adoption in clinical practice.

Deep learning has emerged as a transformative tool for seizure detection and EZ localization, offering significant advantages over traditional methods. However, challenges related to model generalization, interpretability, and real-time deployment must be overcome to enable successful clinical integration. Future research should emphasize cross-institutional validation, multi-modal approaches, and neuromorphic computing solutions to advance AI-driven epilepsy diagnostics. With continued collaboration between machine learning researchers and clinicians, deep learning holds the potential to improve epilepsy care.
